# High-Throughput Identification of Candidate Strains for Biopreservation by Using Bioluminescent *Listeria monocytogenes*

**DOI:** 10.3389/fmicb.2018.01883

**Published:** 2018-08-17

**Authors:** Sara M. El Kheir, Lamia Cherrat, Ahoefa A. Awussi, Nancy E. Ramia, Samir Taha, Abdur Rahman, Delphine Passerini, Françoise Leroi, Jeremy Petit, Cécile Mangavel, Anne-Marie Revol-Junelles, Frédéric Borges

**Affiliations:** ^1^Université de Lorraine, LIBio, Nancy, France; ^2^Laboratoire de Biotechnologies Appliquées, EDST, Université Libanaise, Tripoli, Lebanon; ^3^Department of Industrial Biotechnology, Atta-ur-Rahman School of Applied Biosciences, National University of Sciences and Technology, Islamabad, Pakistan; ^4^Laboratoire Ecosystèmes Microbiens et Molécules Marines pour les Biotechnologies, Ifremer, Nantes, France

**Keywords:** biopreservation, *Listeria monocytogenes*, high-throughput screening assays, bioluminescence, competition, mixed culture, bioprotection, antibacterial activities

## Abstract

This article describes a method for high-throughput competition assays using a bioluminescent strain of *L. monocytogenes*. This method is based on the use of the luminescent indicator strain *L. monocytogenes* EGDe*lux.* The luminescence of this strain is correlated to growth, which make it suitable to monitor the growth of *L. monocytogenes* in mixed cultures. To this aim, luminescence kinetics were converted into a single numerical value, called the Luminescence Disturbance Indicator (LDI), which takes into account growth inhibition phenomena resulting in latency increase, decrease in the luminescence rate, or reduction of the maximum luminescence. The LDI allows to automatically and simultaneously handle multiple competition assays which are required for high-throughput screening (HTS) approaches. The method was applied to screen a collection of 1810 strains isolated from raw cow’s milk in order to identify non-acidifying strains with anti-*L. monocytogenes* bioprotection properties. This method was also successfully used to identify anti-*L. monocytogenes* candidates within a collection of *Lactococcus piscium*, a species where antagonism was previously described as non-diffusible and requiring cell-to-cell contact. In conclusion, bioluminescent *L. monocytogenes* can be used in HTS to identify strains with anti-*L. monocytogenes* bioprotection properties, irrespectively of the inhibition mechanism.

## Introduction

Although constant efforts are being made to ensure food safety, foodborne illnesses remain a major concern in developing and developed countries (Wang et al., 2016). In France, from 2008 to 2013, it is estimated that fifteen foodborne pathogens led to 1.28–2.23 millions ill patients and 250 deaths (Van Cauteren et al., 2017). More specifically, *Listeria monocytogenes* was responsible for approximately 2200 human cases of listeriosis in 2015 in European Union (EFSA, 2016). Food safety is also an important economic issue: in the United States, the annual economic impact of health loss is estimated as more than 77 billions dollars (Scharff, 2012). Apart pathogens, microorganisms can induce food spoilage leading to food waste (Barth et al., 2009; Remenant et al., 2015; Leyva Salas et al., 2017). Reducing food waste is a major challenge since each year 1.3 billions tons of food are lost or wasted (Gustavsson et al., 2011).

Biopreservation, also called bioprotection, is one of the existing strategies to inhibit unwanted microorganisms in food. As it is based on the use of competitive microorganisms (Stiles, 1996; Drider et al., 2006; Elsser-Gravesen and Elsser-Gravesen, 2014; Leyva Salas et al., 2017), a critical step in biopreservation is the selection of antagonistic microorganisms. Usually, in the selection process, the first step of the screening process relies on the detection of inhibitory zones after cultivation in laboratory media (Schillinger and Lucke, 1989; Sobrino et al., 1991; Larsen et al., 1993; Villani et al., 1994; Coventry et al., 1997). Lately, several diffusion methods were still successfully applied, such as the spot-on-lawn (Morales-Estrada et al., 2016), the well-diffusion (Lakshminarayanan et al., 2013; Nami et al., 2015), or the Oxford cup methods (Bian et al., 2016). At the end of this step, the number of candidates is markedly lowered, thus allowing to reduce the workload in the following steps. In the next step, the remaining candidates are used to challenge the growth of targeted microorganisms in food products. These tests are generally performed by using the time-consuming technique of dilution plating and colony counting. Although this approach allows identifying very powerful bioprotective bacteria, it has several drawbacks. Indeed, at the end of the first step, a large majority of microorganisms is discarded. However, even though no or weak inhibition can be recorded in model laboratory media, it cannot be excluded that some of them could exhibit potent antagonistic potential in food. In addition, this method does not take into account inhibition involving non-diffusible compounds. Bacteria can indeed inhibit other bacteria by establishing a direct cell-to-cell contact, as it is exemplified by the anti-listerial property of *Lactococcus piscium* (Saraoui et al., 2016). It is likely that these methodological biases lead to wide underestimation of this type of antagonism. Therefore, it would be more relevant to use the food matrix itself instead of the laboratory medium in the first step of the screening process in order to select potent strains for bioprotection applications. To do so, workflows allowing to performing mixed cultures with high-throughput are required.

High-throughput screening (HTS) methods have been widely used for drug discovery for a long time (Fan and Wood, 2007). Lately, a HTS method was developed to screen *Lactobacillus* species for antibacterial and antifungal activities (Inglin et al., 2015). However, methods based on HTS generally rely on turbidity weakening or changes in absorbance or light scattering properties, and therefore cannot be applied to opaque food extracts (Inglin et al., 2015; Vijayakumar and Muriana, 2015; Liu et al., 2016; Paytubi et al., 2017). Reporter-based assays using fluorescence or bioluminescence then appear to be attractive detection techniques for the development of a HTS method on food-based matrices. Recently, two HTS methods were described for the pathogens *Mycobacterium abscessus* and *Escherichia coli* (Nybond et al., 2013; Gupta et al., 2017). The targeted microorganisms were specifically labeled by genetic-engineering, leading to strains carrying genes encoding for the synthesis of the protein responsible for signal production. Apart from the specificity of the signal, the advantage of fluorescence or bioluminescence detection resides in their high sensitivity (Fan and Wood, 2007; Osterman et al., 2016). A major advantage of bioluminescence over fluorescence reporters such as the green fluorescent protein is the shorter half-life of luciferases. In addition, fluorescent reporters require to be excited prior to measurement, which can result in high background noise due to autofluorescence (Morrissey et al., 2013).

An optimized bioluminescent strain of *L. monocytogenes* was reported (Riedel et al., 2007). This strain, named *L. monocytogenes* EGDe*lux*, was constructed in the recipient strain *L. monocytogenes* EGDe by chromosomal integration of the integration vector pPL2*lux*, which carries a synthetic operon *lux* derived from *Photorhabdus luminescens* and optimized for Gram-positive bacteria (Bron et al., 2006). *L. monocytogenes* EGDe*lux* was successfully detected by luminescence monitoring in various matrices, including broth, mouse body, camembert cheese, hotdog, and meat (Riedel et al., 2007; Morrissey et al., 2013), and therefore would be a good candidate to implement a HTS strategy in the field of biopreservation.

The aim of this study was to establish a HTS strategy based on the use of a bioluminescent strain of *L. monocytogenes* allowing to identify microorganisms exhibiting anti-*Listeria* activity. The relationship between growth inhibition and luminescence was determined, and a data processing method of bioluminescence kinetics was developed. As a proof of concept, this method was applied to screen two bacteria collections, thus allowing to identify candidate strains with anti-*L. monocytogenes* bioprotection properties, irrespectively of the inhibition mechanism.

## Materials and Methods

### Bacterial Strains and Culture Conditions

Microbial cultures were performed in level two containment laboratories. The strain *L. monocytogenes* EGDe*lux* (Riedel et al., 2007) was cultivated in Trypticase Soy Broth medium (TSB) (bioMérieux, Marcy-l’Etoile, France) supplemented with 6 g.L^-1^ of bacto-yeast extract (YE) for 16 h at 30°C or in Brain Heart Infusion medium (BHI) supplemented with 2% NaCl (BIOKAR Diagnostic, Beauvais, France) for 24 h at 26°C. The strain *L. monocytogenes* RF191 was isolated from tropical cooked peeled shrimp by PFI Nouvelles Vagues (Boulogne-sur-Mer, France) and used as reference strain to screening protective culture for fish products (Fall et al., 2010; Saraoui et al., 2016). *L. monocytogenes* RF191 was cultured in BHI medium supplemented with 2% NaCl (BIOKAR Diagnostic, Beauvais, France) for 24 h at 26°C. Strains were stored in TSBYE or BHI medium supplemented with glycerol (10%) at -80°C and propagated twice before use.

A collection of 1810 strains was built by isolating bacteria from 84 milk samples originating from the north of France. Milk samples were serially diluted in saline (9 g.L^-1^ NaCl) and 100 μL of each suspension was plated on Medium for *Carnobacterium maltaromaticum* (MCM) medium (Edima et al., 2007). Colonies were picked and transferred into 96-well plates filled with TSBYE medium for cultivation at 30°C during 24–48 h prior to storage at -80°C. These 96-well microplates were thawed and used to inoculate commercial UHT cow whole milk medium in 96-well microplate, which was incubated at 30°C for 48 h prior to screening assays.

*Lactococcus piscium* strains were isolated from cold-stored packaged foods such as seafood products (Ifremer/Oniris, Nantes, France) and meat products (ADIV, France). The 41 strains of *L. piscium* were stored at -80°C in Elliker medium (BIOKAR Diagnostic, Beauvais, France) with a final concentration of 20% glycerol (v/v).

### Luminescence Measurement and Definition of Luminescence Disturbance Indicator

Luminescence was measured over time in a microplate reader Infinite 200Pro equipped with a luminescence module (Tecan Group Ltd., Männedorf, Switzerland) in white opaque 96-well microplates. Turbidity was also measured in parallel with the same equipment by recording Optical Density (OD) at 595 nm using transparent 96-well microplates. The Luminescence Disturbance Indicator (LDI), was defined as the inverse of the harmonic mean of the ratio between luminescence and delay time, and calculated as follows:

$$LDI=\frac{1}{n}\sum \frac{l}{t}$$

where *n* is the number of timepoints, *l* the luminescence expressed as photon counts, and *t* the delay time (sec). Thus, LDI unit is photons count.sec^-1^.

The indicator of luminescence inhibition LDI_r_ was obtained as follows:

$$LDIr=100\times \frac{LDI}{LDIc}$$

where LDI is a value obtained in one given condition and LDI_c_ a value obtained in a control experiment where no inhibition occurs.

### Impact of Adverse Growth Conditions

The strain *L. monocytogenes* EGDe*lux* was cultivated in TSBYE media at different pH values (from pH 4.5 to 9 by 0.5 steps) as well as in TSBYE previously supplemented with NaCl (20, 40, 60, and 80 g.L^-1^) or nisin (Sigma N5764; 0.00125, 0.005, 0.01, 0.04 g.L^-1^). OD_595__nm_ and luminescence were recorded over time and LDI values were subsequently calculated.

### Identification of Strains Isolated From Raw Milk Inhibiting the Luminescence of *L. monocytogenes* EGDe*lux*

The 1810 strains of the collection, which were previously cultivated in UHT whole cow’s milk, were serially diluted 10^5^ times in UHT whole cow’s milk in order to reach 200 μL final volume. The microplates containing diluted strains were used for the co-culture experiment with *L. monocytogenes* EGDe*lux.* Prior to inoculation with *L. monocytogenes*, these microplates were incubated for 2 h at 30°C. In parallel, the strain *L. monocytogenes* EGDe*lux* was subcultured in 10 mL TSBYE medium at 20°C for 24 h, then washed twice by centrifugation at 5000 *g* for 2 min, and the cells were resuspended in 10 mL sterile water. This suspension was diluted 1000 times in UHT whole milk. Twenty microliters of this *L. monocytogenes* EGDe*lux* suspension was used to inoculate each well of the microplates after 2 h incubation at 30°C. The co-cultures were incubated at 30°C for 16 h and luminescence of the co-cultures was measured every 40 min. Incubation and luminescence monitoring were performed with a microplate reader Infinite 200Pro equipped with a luminescence module (Tecan) coupled to an automated liquid handling and incubation system (Tecan).

### Identification of Non-Acidifying Microorganisms

Each strain of the raw milk microorganisms collection was cultivated in 200 μL of milk supplemented with 0.0035% (w/v) bromocresol purple in transparent 96-well microplates at 30°C for 24 h. Non-acidifying microorganisms were identified by visual inspection of the microplate cultures, by considering that bromocresol purple is purple above pH 6.8 and yellow below pH 5.2.

### Bacterial Enumeration by Serial Micro-Dilutions and Colony Counting

Serial dilutions were performed in 96-well microplates by filling each well with 180 μL of saline and transferring 20 μL culture test by using a Freedom evo automated liquid handling system (Tecan). Palcam medium in square plates was inoculated with 2.5 μL of dilution with the automated liquid handling system. After incubation at 37°C for 24–36 h, the colonies were counted and the concentration of *L. monocytogenes* was subsequently calculated.

### Identification of *L. piscium* Strains With Anti-*L. monocytogenes* Properties

The sensitivity of *L. monocytogenes* EGDe*lux* to 41 *L. piscium* strains was compared to the sensitivity of the *L. monocytogenes* RF191 reference strain. The RF191strain was isolated from tropical cooked peeled shrimp by PFI Nouvelles Vagues (Boulogne-sur-Mer, France) and used as reference strain to screen protective culture for fish products in the chemically defined MSMA medium previously described (Fall et al., 2010; Saraoui et al., 2016). The strains *L. monocytogenes* RF191 and *L. monocytogenes* EGDe*lux* were subcultured in 10 ml of BHI supplemented with 2% NaCl (BIOKAR Diagnostic, Beauvais, France) for 24 h at 26°C. For the screening assays, each *L. piscium* strain was co-cultured with *L. monocytogenes* EGDe*lux* or RF191 strains in 200 μL of MSMA medium (Saraoui et al., 2016), in a sterile 96-well microplate, at initial concentrations of approximately 10^6^ CFU.mL^-1^ for *L. piscium* and 10^3^ CFU.mL^-1^, for *L. monocytogenes*. The inhibition of the strain *L. monocytogenes* EGDe*lux* was quantified by measuring the luminescence every 30 min during 50 h using the GENios Plus Luminescence Microplate reader (Tecan) followed by the calculation of LDI_r_. The inhibition of the *L. monocytogenes* RF191 strain was measured by enumeration at 30 or 60 h by serial micro-dilution and colony counting. The inhibition was quantified by comparing the growth of *L. monocytogenes* in co-culture with *L. piscium* strains and in pure culture.

## Results

### Suitability of *L. monocytogenes* EGDe*lux* to Reveal Growth Inhibition

**FIGURE 1:**
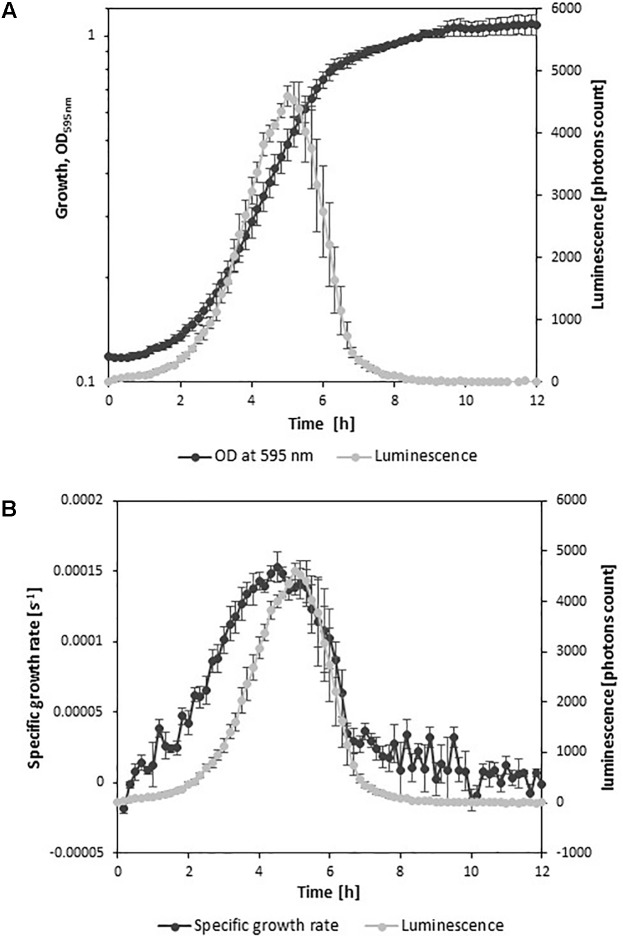
Growth kinetics and luminescence of *L. monocytogenes* EGDe*lux*. **(A)** OD_595__nm_ and luminescence. **(B)** Growth rate and luminescence. Error bars represent standard errors.

Cultivation of the strain *L. monocytogenes* EGDe*lux* in TSBYE broth revealed that the major production of luminescence occurred during the exponential growth phase (**Figure 1A**). The evolution of luminescence was well correlated to the evolution of the specific growth rate (**Figure 1B**). This strongly suggests that the production of luminescence reflects the intensity of the metabolic activity of *L. monocytogenes*.

**FIGURE 2:**
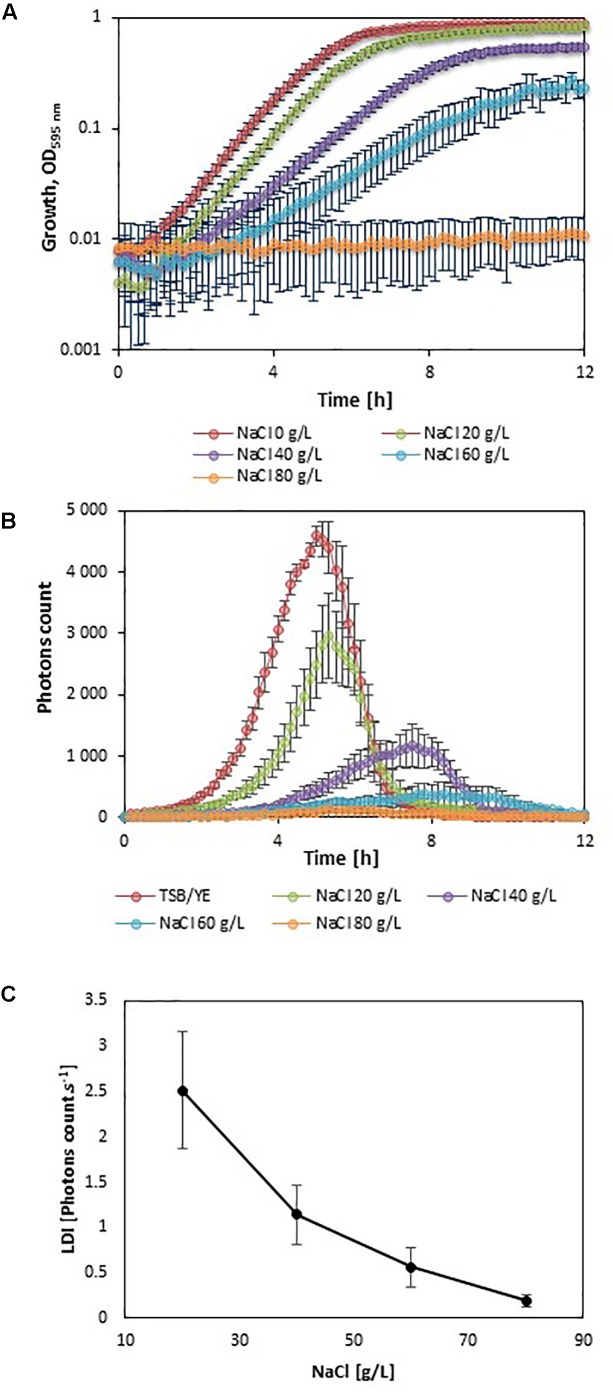
Impact of NaCl concentration on growth and luminescence of *L. monocytogenes* EGDe*lux.* **(A)** biomass production kinetics, **(B)** luminescence kinetics, and **(C)** evolution of LDI with NaCl concentration. Error bars represent standard errors.

**FIGURE 3:**
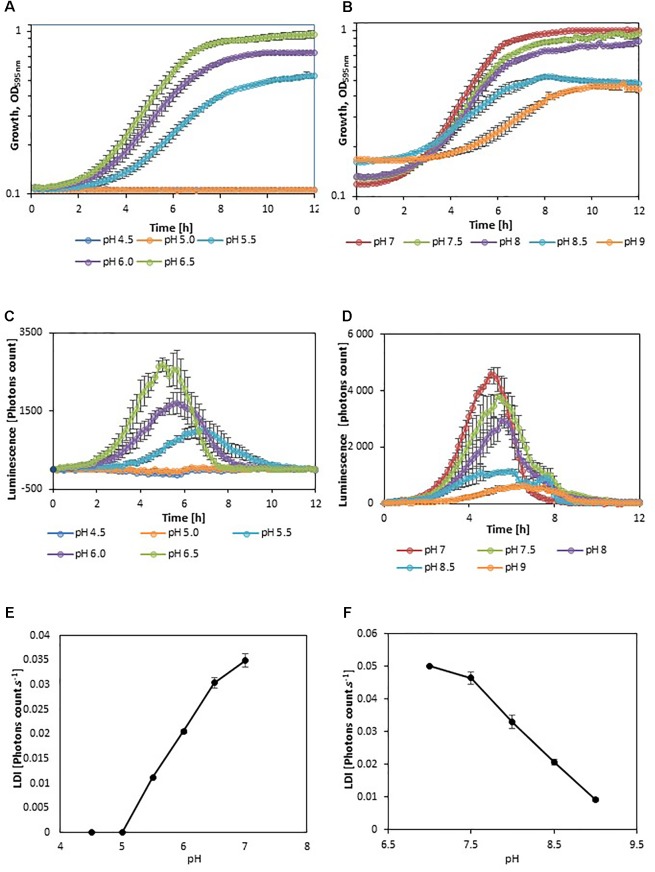
Impact of pH on growth and luminescence of *L. monocytogenes* EGDe*lux*. **(A,D)** biomass production kinetics, **(B,E)** luminescence kinetics, and **(C,F)** evolution of LDI with pH. **(A–C)** acidic pH values and **(D–F)** basic pH values. Error bars represent standard errors.

**FIGURE 4:**
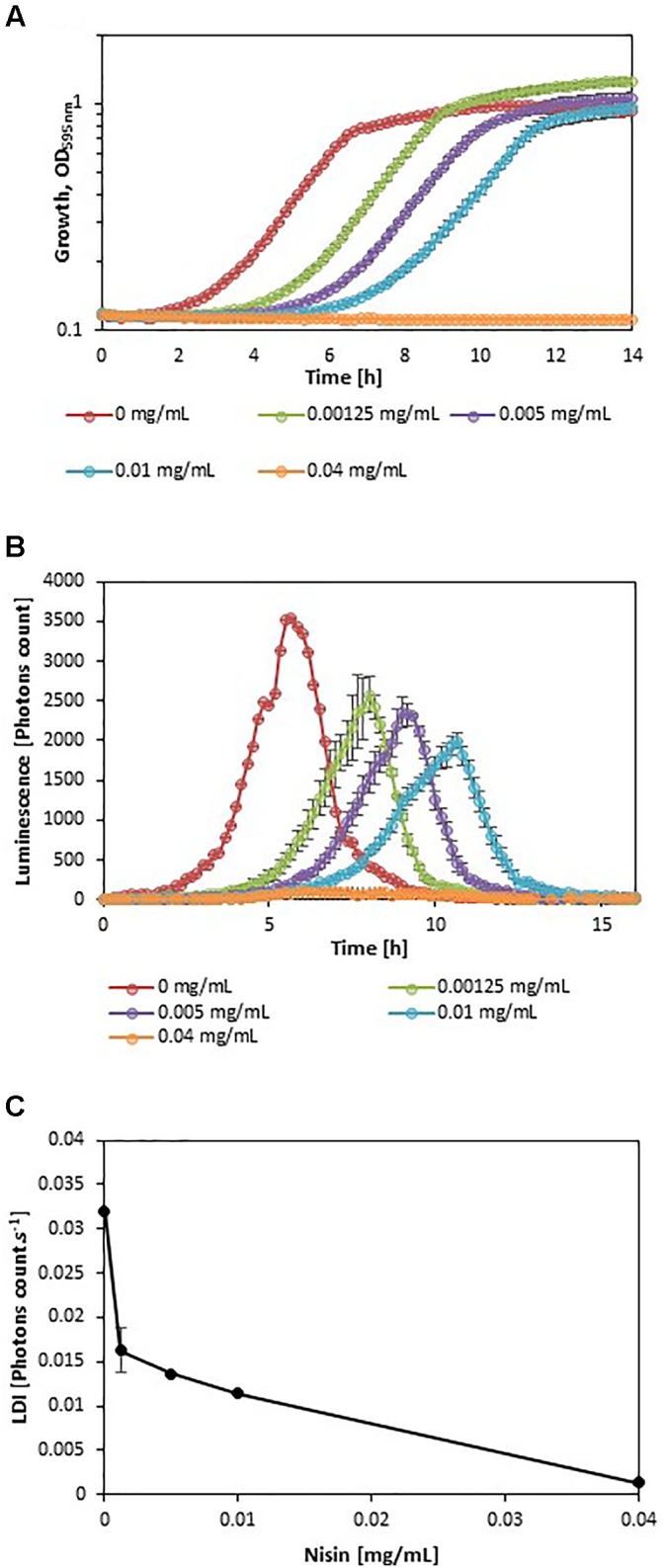
Impact of nisin concentration on growth and luminescence by *L. monocytogenes* EGDe*lux.* **(A)** biomass production kinetics, **(B)** luminescence kinetics, and **(C)** evolution of LDI with nisin concentration. Error bars represent standard errors.

In order to evaluate the impact of adverse growth conditions on luminescence, *L. monocytogenes* EGDe*lux* was cultivated in different conditions of salt concentration, pH, and nisin concentration. A higher NaCl concentration caused decreases in the specific growth rate and final biomass production at stationary phase (**Figure 2A**). Both the rate of luminescence and the maximum of luminescence decreased (**Figure 2B**). Similar results were obtained when different pH values were investigated (**Figures 3A,B,D,E**). When nisin concentration was increased, an increase of the latency and of biomass production was recorded (**Figure 4A**) and the production of bioluminescence was also delayed (**Figure 4B**). However, when *L. monocytogenes* EGDe*lux* was cultivated with 0.04 mg.mL^-1^ nisin, neither growth nor luminescence was observed. The results show that kinetics of luminescence reflects the growth behavior; and hence that luminescence reduction can be used as an indicator of *L. monocytogenes* growth inhibition.

### Transposition to High-Throughput Screening Thanks to the Luminescence Disturbance Indicator

**FIGURE 5:**
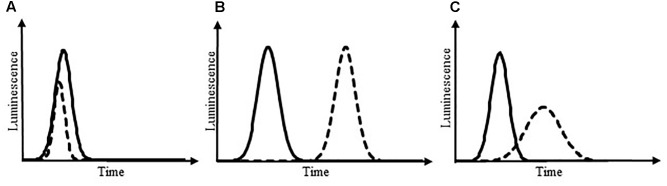
Luminescence kinetics under different scenarii of growth inhibition. Solid line: control; dotted line: sample exhibiting luminescence changes owing to growth inhibition. Growth inhibition can lead to a reduced pic size of luminescence **(A)**, a delayed pic **(B)**, or a pic with decreased slopes **(C)**. In these theoretical scenarii, the LDI_r_ is of 50%.

Using luminescence kinetics (temporal evolution of luminescence) in order to monitor *L. monocytogenes* growth inhibition in a HTS approach cannot be envisaged owing to the complexity of data handling; it is indeed difficult to compare dozens of curves simultaneously. Therefore, in the present work, a data processing strategy was devised in order to characterize each kinetic curve by a single parameter representative of luminescence inhibition features. Thus, this parameter must take into account the multiple typologies of *L. monocytogenes* growth inhibition: the maximum luminescence can be delayed, the rate of luminescence can be decreased, and/or the maximum of luminescence can be lowered. At first glance, the integral of luminescence was viewed as a potential good candidate, as it can reveal growth inhibitions resulting in a reduction of the overall luminescence. However, the discrete integral (sum) of luminescence cannot evidence delayed luminescence, all other luminescence characteristics being equal, as the time factor is erased by application of the integral. Therefore, the building of the parameter representative for luminescence delay was then improved by weighting the luminescence with the inverse of time. Following this approach, the higher the delay of luminescence, the lower the obtained value of the parameter representative for luminescence. Finally, the discrete integral of luminescence weighted by time was divided by the number of recorded experimental points in order to get a final parameter representative for luminescence that is independent from measurement frequency. This leads to the LDI which is the inverse of the harmonic mean of the ratio between luminescence and time. By definition, the LDI is thus a positive real number that decreases when growth is more inhibited. According to the following formula:

$$LDI=\frac{1}{n}\sum \frac{l}{t}$$

the LDI of a sample exhibiting growth inhibition is inferior to the LDI of a reference sample in the different inhibition scenarii presented in **Figure 5**. In these scenarii, for which the LDI_r_ is of 50%, the growth inhibition can lead to three different typologies: the maximum pic size can be reduced (**Figure 5A**), the pic can be delayed (**Figure 5B**) or the pic can be characterized by a decreased slope (**Figure 5C**). Thus, the LDI is able to reveal luminescence inhibition irrespective of the inhibition typology.

The LDI was calculated with the data obtained from the experiments where *L. monocytogenes* EGDe*lux* was cultivated under different conditions of pH, NaCl concentration, and nisin concentration (**Figures 2C**, **3C,F**, **4C**). The results revealed that the LDI decreased when the concentration of NaCl and nisin were increased. Similarly, the LDI decreased when pH deviated from neutrality. These results show that LDI mirrored the growth of *L. monocytogenes* EGDe*lux* and can be used for data handling of luminescence kinetics in HTS approaches.

### Identification of Anti-*Listeria* Activity Within a Collection of Raw Milk Bacteria by High-Throughput Screening

To illustrate the use of bioluminescent *L. monocytogenes* for HTS purpose, a screening was carried out, which aimed at identifying candidate strains with anti-*L. monocytogenes* properties for biopreservation applications. The cheese industry being targeted, a collection of microorganisms isolated from raw milk was constructed and a multistep screening strategy was developed in order to fulfill two main criteria: (i) the strains should exhibit anti-*Listeria* properties whatever the inhibitory mechanism involved, and (ii) the strains should not interfere with the acidification step of the cheese making process.

The screening was performed with a collection of 1810 microorganisms (**Supplementary Table S1**). Each strain of the collection was co-cultivated with the strain *L. monocytogenes* EGDe*lux* in UHT cow’s whole milk. The kinetics of luminescence was used to calculate LDI and LDI_r_ values. LDI_r_ is the percentage of the remaining luminescence and is calculated by considering the LDI obtained in a given test condition (*i.e.*, when *L. monocytogenes* EGDe*lux* was co-cultivated with a candidate strain) and the LDI value obtained in the control experiment (*i.e., L. monocytogenes* EGDe*lux* in pure culture). This HTS resulted in LDI values ranging from 3.5 × 10^-3^ to 4.2, with 17% of the strains (318 strains) resulting in LDI_r_ values below 50%, indicating that they inhibited the production of luminescence by at least 50% (**Supplementary Table S2** and **Figure S1**).

This subset of 318 strains was selected to continue the screening. The next step was designed in order to identify non-acidifying strains among this subset. Indeed, the use of protective cultures in cheese manufacturing should not interfere with the acidification step. In line with this, the subset of 318 strains was screened for their inability to acidify milk. Among the 318 strains, 240 strains exhibited acidifying properties (**Supplementary Table S3**).

The remaining 78 non-acidifying strains, which represent 4% of the initial collection, were screened for their ability to inhibit *L. monocytogenes* EGDe*lux* by performing mixed-cultures in 96 microplates and determining the concentration of *L. monocytogenes* by using a method of serial micro-dilution and colony counting after drop spotting. This step allowed to delineate 55 strains able to inhibit the growth of *L. monocytogenes* by at least 35 times (**Supplementary Table S4**), corresponding to at least 1.5 log reduction. Among them, 9 strains could inhibit *L. monocytogenes* by at least up to 100 times, *i.e.*, 2.6 log reduction. Furthermore, it can be noticed that the 78 strains were tested for their ability to inhibit *L. monocytogenes* by applying the classical agar diffusion-based method as well (data not shown). This method revealed that only two strains could inhibit *L. monocytogenes* by producing an inhibition halo. In addition, these two strains were not found among the strains with the highest inhibition potency according to the luminescence-based screening.

These results show that beyond selecting candidate strains with anti-*L. monocytogenes* properties, the use of luminescence-based screening allowed the selection of potent anti-*L. monocytogenes* strains that the classical selection procedure would have failed to detect because it is based on the detection of diffusible antagonistic compounds.

### High-Throughput Screening of Non-diffusible Anti-*Listeria* Activity

The strain *L. piscium* CNCM I-4031 is described to inhibit the growth of *L. monocytogenes* when both microorganisms are co-cultivated (Saraoui et al., 2016). Although the molecular mechanism of inhibition is still unknown, it is described as resulting from a compound that is not secreted in the medium as it would be in the case for bacteriocins, organic acids, or hydrogen peroxide (Saraoui et al., 2016). It was proposed that the inhibition of *L. monocytogenes* by *L. piscium* CNCM I-4031 (formerly EU2241) requires cell-to-cell contact (Saraoui et al., 2016). Therefore, the detection of this inhibitory activity is time-consuming, as a simple agar diffusion assay fails to reveal any inhibition of *L. monocytogenes*. Indeed, it requires to perform co-cultures in liquid medium and cell enumeration. Serial dilutions and plating for colony counting is not easy to scale up to high throughput format.

The suitability of using *L. monocytogenes* EGDe*lux* and the LDI to characterize the anti-*Listeria* activity of a strain collection of *L. piscium* was assessed. First, the inhibition of *L. monocytogenes* EGDe*lux* by the strain *L. piscium* CNCM I-4031 was assessed and compared with the reference strain *L. monocytogenes* RF191 generally used to quantify the *L. piscium* activity in fish products (Fall et al., 2010; Saraoui et al., 2016). After 30 h of co-cultures with *L. piscium* CNCM I-4031 in MSMA medium, both *Listeria* strains were enumerated by serial micro-dilution and colony counting after drop spotting, in four replicates. The EGDe*lux* showed an inhibition of 1.5 ± 0.7 log CFU.mL^-1^ when compared to the pure culture. Although the inhibition was lower than for the reference strain RF191 which exhibited in the same conditions an inhibition of 3.8 ± 0.2 log CFU.mL^-1^, the use of EGDe*lux* as indicator strain was validated to select new bioprotective strains.

The effect of 41 strains of *L. piscium* including CNCM I-4031 on *L. monocytogenes* was analyzed by two screening methods based (i) on the luminescence production of *L. monocytogenes* EGDe*lux* and (ii) on the growth of *L. monocytogenes* RF191 calculated by micro-enumeration at 60 h (**Supplementary Table S5**). The results revealed that 33 strains inhibited *L. monocytogenes* EGDe*lux* according to the LDI_r_ values, and 30 strains inhibited the growth of the *L. monocytogenes* RF191. These results suggested a large proportion (approximately 80%) of *L. piscium* strains were able to inhibit *L. monocytogenes* but with variable intensity. The reference strain *L. piscium* CNCM I-4031 was ranked fourth for the inhibition of *L. monocytogenes* RF191 (1.19 log CFU.mL^-1^) and ranked seventh for the inhibition of *L. monocytogenes* EGDe*lux* (LDI_r_ 0.71). Interestingly, the strains *L. piscium* MIP2407 and MIP2450 showed better anti-listerial activity than the strain *L. piscium* CNCM I-4031 with the two screening methods. These two strains showed an inhibition over 1.3 log UFC.mL^-1^ and a LDI_r_ under 0.6. On the contrary, some strains were exhibit more potent inhibitoy properties than *L. piscium* CNCM I-4031 for one of the two *L. monocytogenes*. Three strains (*L. piscium* EU2229, EU2230, and EU2231) highly inhibited the growth of *L. monocytogenes* RF191 but not *L. monocytogenes* EGDe*lux*; and four strains (*L. piscium* ADIV9, ADIV36, MIP2572, and MIP2482) highly inhibited the growth of *L. monocytogenes* EGDe*lux* but not *L. monocytogenes* RF191. Overall, these results show that although the sensitivity level of the two strains of *L. monocytogenes* were different, the luminescent strain *L. monocytogenes* EGDe*lux* allowed to efficiently detect *L. piscium* strains with anti-*L. monocytogenes* properties.

## Discussion

The production of bioluminescence by *L. monocytogenes* EGDe*lux* is closely related to the specific growth rate of the bacteria. Indeed, it was described that bioluminescence is highly dependent on the availability of ATP, which is present at high concentrations in growing bacteria (Meighen, 1993). It was also previously described that the maximum of luminescence occurred during the exponential growth phase (Riedel et al., 2007). The strain *L. monocytogenes* EGDe*lux* was cultivated under adverse conditions of NaCl and nisin concentrations, as well as acidic and basic pH. All these conditions are known to impair the growth of *L. monocytogenes* in a dose-dependent manner. It was evidenced in the current study that they also had a respective impact on bioluminescence, highlighting a strong correlation between growth and bioluminescence.

Kinetics data are not convenient for high-throughput analyses. That is why the use of bioluminescence at high-throughput scale requires a powerful data handling tool that would allow to easily automatize data analyses. However, this mathematical tool had to take into account the different possible inhibition patterns: the production of luminescence can be delayed, the rate of luminescence can be decreased, and the maximum of luminescence can be lowered. The LDI was designed to reveal all three types of inhibitions. The LDI was significantly higher than 0, when the NaCl concentration remained below 8%, as well as when pH ranged between 5.5 and 9. Although these physicochemical conditions encompass a wide range of food products, some foods, in particular unripened cheeses, will require pH adjustment prior to using this bioluminescence-based mathematical tool.

This method was successfully applied to screen a collection of 1810 raw milk bacteria and allowed to select 318 strains with potent anti-*L. monocytogenes* activity. Among these 318 strains, 78 strains did not produce acidification of UHT milk. Among them, only two strains produced inhibitory halos in agar diffusion assays. This indicates that if the same collection of bacteria would have been screened by using a classical screening approach based on the production of inhibitory halos, 76 strains would probably have been discarded during the first stages of the classical screening process. The inhibitory activity of these strains might involve non-diffusible antagonistic compounds that may require cell-to-cell contact inhibition as it is described for *Escherichia coli* (Aoki et al., 2005) and *Streptococcus pneumoniae* (Guiral et al., 2005).

The assessment of 41 *L. piscium* strains for their anti-listerial properties in order to select potential bioprotective agent for fish products was performed. Six strains displaying better activity than the reference strain *L. piscium* CNCM I-4031 was highlighted by the luminescence HTS with *L. monocytogenes* EGDe*lux* as indicator strain. Among them, two strains exhibited a higher inhibition potency of *L. monocytogenes* RF191 when compared to the strain *L. piscium* CNCM-4031. Moreover, four strains showed an interesting inhibition potential of *L. monocytogenes* EGDe*lux* but not of *L. monocytogenes* RF191 strain. This example showed whereas the use of two indicator strains improve the pool of positive strains, the luminescence HTS alone allows the selection of the best candidates for bioprotection in food products.

During the course of the raw milk collection screening, several strains induced an increase of the LDIr value indicating that in these cases, the bioluminescence of *L. monocytogenes* EGDe*lux* was higher than in the control. These results suggest that the candidate strains promoted the growth of *L. monocytogenes*. Future studies are required to better understand this interesting promoting activity and assess the possible implications for food ecology and food safety.

This study allowed to establish a HTS method based on the use of the bioluminescent strain *L. monocytogenes* EGDe*lux* for the identification of candidate strains for biopreservation applications. This promising method will likely be helpful for not only the identification of inhibitory strains exhibiting anti*-Listeria* properties but also for the identification of strains exhibiting unusual inhibitory mechanisms. In addition, this method could also be used for the study of biotic interactions involving *L. monocytogenes* in diverse matrices including food.

## Author Contributions

DP, FL, JP, CM, A-MR-J, and FB designed the experiments. SEK, LC, AA, NR, AR, DP, and FB performed the experiments. SEK, LC, AA, DP, and FB analyzed the data. SEK, ST, DP, JP, CM, A-MR-J, and FB wrote the manuscript. FB coordinated the study.

## Conflict of Interest Statement

The authors declare that the research was conducted in the absence of any commercial or financial relationships that could be construed as a potential conflict of interest.
